# Comparison of fluoxetine and duloxetine hydrochloride therapeutic effects on patients with constipation-predominant irritable bowel syndrome 

**Published:** 2022

**Authors:** Sattar Jafari, Behnam Sajedi, Maryam Jameshorani, Fatemeh Salarpour

**Affiliations:** 1 *department of internal medicine, Zanjan University of Medical Sciences, Zanjan, Iran *; 2 *Shahid Beheshti University of Medical Sciences, Department of Hematology and Blood Banking Tehran, Iran*

**Keywords:** Fluoxetine, Duloxetine hydrochloride, Irritable bowel syndrome, Constipation

## Abstract

**Aim:**

As few randomized clinical trials have verified the efficacy of selective and norepinephrine reuptake inhibitors in IBS, the current study made an inclusive comparison between them, and their effectiveness in IBS-C was proven.

**Background:**

Irritable bowel syndrome with constipation (IBS-C) is a functional bowel disorder characterized by changes in bowel movements and abdominal pain in the absence of identifiable structural abnormalities. Despite much progress in the treatment of other types of IBS, limited treatments are available for IBS-C.

**Methods:**

The study population comprised 182 IBS-C patients who were randomly divided into 3 groups according to treatment type. One group was given 20 mg of dicyclomine and fluoxetine, the second group received dicyclomine along with duloxetine hydrochloride, and the third group received dicyclomine only for two months. The severity of symptoms was recorded by questionnaire at the beginning and end of the treatment.

**Results:**

The average age and BMI of the patients were 28.5 ± 5.2 years and 25.2 ± 2.4 kg/m2, respectively. Duloxetine was more effective than fluoxetine in reducing flatulence (*p*=0.043), abdominal pain intensity (*p*≤0.046), and duration (*p*≤0.003), in increasing the quality of life (*p*≤0.046), and the frequency of fecal excretion in patients (*p*≤0.004).

**Conclusion:**

Based on the study findings, fluoxetine and duloxetine had greater therapeutic effects on all symptoms of IBD than dicyclomine, with duloxetine, specifically, being more effective than fluoxetine. Further studies on larger groups are suggested to determine the best dosage and identify any potential side effects of these drugs.

## Introduction

 Irritable bowel syndrome with constipation (IBS-C) is a chronic intestinal disorder distinguished by pain, altered bowel movement, and often accompanied by severe impairment in the quality of life (QoL), yet it is not a life-threatening condition (1, 2). 

Although IBS-C poses a pressing public health problem, its treatment and diagnosis are still common concerns with clinicians. That might stem from the fact that many healthcare professionals are unaware of how much centrally targeted medications such as antidepressants, which have recently been used in painful disorders and have abnormal regulation in IBS, could be of assistance in IBS patients. In the same vein, several studies have discovered that more than 55% of IBS-C patients were cured by antidepressants. There is no consensus, however, on whether prescribing antidepressants such as tricyclic antidepressants (TCAs), serotonin/norepinephrine reuptake inhibitors (SNRIs), and selective serotonin reuptake inhibitors (SSRIs) have favorable effects on IBS-C per se (3, 4).

SSRIs act mainly by hindering the reuptake of 5-HT, then causing a reduction in back and neuropathic pain, and relieving some GI-specific symptoms and well-being on a large scale. Drugs like fluoxetine could be more beneficial in patients with constipation than in those with diarrhea, as generally, diarrhea is a side effect of such drugs. On the other hand, SNRIs (duloxetine hydrochloride in this study) affect both descending 5-HT and NE pain inhibition systems, and their most common side effects are constipation and dry mouth. Comparatively, SSRIs showed slower antidepressant effects than SNRIs in clinical treatments, probably because of the blockage of norepinephrine reuptake in SNRIs (5, 6).

Arguably, what type of antidepressant drug is efficient and tolerable enough in different kinds of IBS has not yet been elucidated. Nonetheless, TCAs, for example, seem not to be as beneficial in IBS-C as they are in IBS with diarrhea (IBS-D); in contrast, they worsen constipation in these patients (7-9). Given these circumstances, a comprehensive study of the effects of these drugs on IBS-C needs to be undertaken.

Given that duloxetine hydrochloride has obvious efficiency in treating other pain syndromes (10, 11), it might represent a suitable substitute to SSRIs and TCAs for IBS. Hence, the authors accomplished a clinical trial study with 182 IBS-C individuals who had no apparent depressive disorder to investigate this theory in IBS-C and then put forward the best cure. 

## Methods

**Table1:** Diagnostic Rome III criteria for IBS-C

Recurrent abdominal pain or discomfort at least 3 days/month in the last 12 weeks, with two or more of the following three characteristics	
1	pain relieved by defecation
2	The onset of symptoms is associated with alternation in the frequency of stool
3	The onset of symptoms is associated with alternation in the form of stool
Criteria must be present for at least 3 months, and symptoms must have started at least 6 months before diagnosis.	

**Data Collection**

Among patients who referred to the general or gastrointestinal clinic of the hospital, 182 (over the age of 18) with IBS-C were included in this clinical trial study (Table 1). All patients with warning signs such as nocturnal diarrhea, weight loss, gastrointestinal bleeding, or those taking medication (a history of other antidepressant medications such as TCAs or other SSRIs), having any depression problems, drinking alcohol, and consuming narcotic drugs were excluded from the study. Moreover, patients showing any signs or symptoms of organic disease in their history or physical examination and a high level of anxiety interfering with study results were also excluded as were patients who took medicines (sodium bicarbonate, potassium chloride, and sodium chloride) that interfere with dicyclomine or had dangerous underlying heart problems (chronic heart failure, sinoatrial block, and low blood pressure) or severe renal or hepatic failure.

**Study procedures**

After obtaining the approval of the ethics committee (ethic number: IRCT20171115037478N1) and patients’ informed consent, participants were randomly divided into three disparate groups using computerized randomizing tables. Sixty-one participants received 20 mg per day fluoxetine along with dicyclomine (Pars Darou Company); another 61 patients were given 20 mg per day duloxetine hydrochloride along with dicyclomine, and the rest took dicyclomine only (as a conrol group) (Figure 1). These open-label and random-table interventions were done for two months. At the onset and two months after conclusion of the study, patients were followed-up and grouped in four categories according to IBS-symptom severity scale scores (IBS < 75 = no IBS, 75-175 = mild IBS, 175-300 = moderate IBS, and 300 = severe IBS).

**Sample size calculation and statistical analysis**

The sample size was evaluated with the formula n= $\frac{{\left[Z_{1-\frac{a}{2}}+Z_{1-\beta }\right]}^{2}\left[\delta^{2}\right]}{{\left(\mu 1-\mu 2\right)}^{2}}$

Data was analyzed using SPSS 16.0 (SPSS Japan, Tokyo), and data was expressed as mean ± standard deviation in the tables. A *p-*value ≤0.05 was considered significant. Shapirovik-Wilk and Kolmogorov-Smirnov tests were used in the normal distribution of data; however, T-test and ANOVA were applied when the data showed no normal distribution. The Pearson test was used to measure any correlation between the three studied drugs. 

**Table2:** The effects of the studied drugs on the intensity of patients' abdominal pain

Variable		Fluoxetine		Duloxetine Hydrochloride		Dicyclomine		p-value Fluoxetine and Duloxetine Hydrochloride
		Number	Percentage	Number	Percentage	Number	Percentage	0.045 0.001
Abdominal pain before intervention	Very intense	11	18	10	16.4	11	15	
	intense	25	41	27	44.3	23	45	
	More or Less	25	41	21	34.4	20	37.5	
	painless	0	0	3	4.9	6	2.5	
Abdominal pain after intervention	Very intense	0	0	1	1.6	11	3/18	
	intense	12	19.7	4	6.6	10	7/16	
	More or Less	34	55.7	27	44.3	14	3/23	
	painless	15	24.6	29	47.5	25	7/41	
	p-value	0.002		<0.0001		0.066		
	p-value	0.046						

## Results

The current clinical trial study was carried out on 182 patients (105 female and 77 male) with irritable bowel syndrome with constipation (IBS-C). These patients were divided into three groups; 61 patients received the fluoxetine and dicyclomine intervention, 61 patients received the duloxetine hydrochloride and dicyclomine intervention, and the remaining participants received dicyclomine only. The mean ± SD of their age was 28.80±5.92 (range from 18 to 40 years), and their average *Body mass index* (*BMI*) was 25.45±2.19 (between 21 and 33). Fifty-eight participants were female, while the percentage of males was 41.4%. More than half of the participants were married (57.4%), and the rest were single (42.6%). There was no significant association between gender or age and prescribing the two studied drugs.

**Duloxetine hydrochloride has a greater effect on reducing the intensity of patients’ abdominal pain than fluoxetine**

Fluoxetine was able to significantly reduce the severity of patients’ abdominal pain (*p*≤0.002); similarly, duloxetine hydrochloride had the same effect (*p*≤0.001). Compared to fluoxetine, however, the effects of duloxetine hydrochloride were greater in these patients (*p*≤0.046). Patients who received dicyclomine showed the drug had no significant impact on such pain (*p*≤0.066) (Table 2).

**Duloxetine hydrochloride has more lessening influence on patients’ flatulence than fluoxetine**

Fluoxetine was able to significantly decrease flatulence in patients (*p*≤0.043). Duloxetine hydrochloride, likewise, had the same effect (*p*≤0.001). A comparison of the two drugs revealed that duloxetine hydrochloride had a greater impact than fluoxetine (*p*≤0.001), but the flatulence rate was insignificant before intervention between the two groups (*p*≤0.351). Nevertheless, dicyclomine did not affect flatulence notably (Table 3).

**Duloxetine hydrochloride has a greater impact on decreasing the duration of patients’ abdominal pain than fluoxetine**

Even though fluoxetine and duloxetine hydrochloride both significantly reduced the duration of pain in patients (*p*≤0.002 and *p*≤0.001), respectively), duloxetine hydrochloride had a notably greater impact in comparison to fluoxetine (*p*≤0.003). Conversely, dicyclomine only slightly affected the duration of abdominal pain (*p*≤0.048) (Table 4).

**Duloxetine hydrochloride has a greater influence on improving the disruption in patients’ lifestyle than fluoxetine**

Even though both fluoxetine and duloxetine hydrochloride caused a significant decline in the rate of life disruption in patients (*p*≤0.046) and (*p*≤0.001), duloxetine hydrochloride had a much greater effect on such issue than fluoxetine (*p*≤0.009). Conversely, dicyclomine played no role in altering such situation (*p*≤0.093) (Table 5).

**Duloxetine hydrochloride has a greater influence on patients’ defecation frequency in comparison to fluoxetine**

Despite the fact that duloxetine hydrochloride more significantly increased defecation frequency in IBD-C patients than fluoxetine (*p*≤0.004), both drugs were similarly able to increase the number of defecations in these patients, with *p*≤0.043 in duloxetine hydrochloride and *p*≤0.001 in fluoxetine. The effect of duloxetine, by contrast, was not significant in such item (*p*≤0.526) (Table 6).

**Table 3:** The effects of the studied drugs on flatulence

Variable		Fluoxetine		Duloxetine Hydrochloride		Dicyclomine		p-value Fluoxetine and Duloxetine Hydrochloride
		Number	Percentage	Number	Percentage	Number	Percentage	0.351 0.001
Flatulence before intervention	Very intense	7	11.5	9	14.8	10	12.5	
	intense	32	52.5	41	67.2	25	50.0	
	More or Less	20	32.8	10	16.4	17	30.0	
	without	2	3.3	1	1.6	8	7.5	
Flatulence after intervention	Very intense	3	4.9	0	0	25	47.7	
	intense	12	19.7	11	18.0	17	27.7	
	More or Less	28	45.9	34	55.7	13	17.7	
	without	18	29.5	16	26.2	5	9.9	
	p-value	0.043		<0.0001		0.359		
	p-value	0.0436						

**Table 4:** The effects of the studied drugs on the duration of patients’ abdominal pain

Variable		Fluoxetine		Duloxetine Hydrochloride		Dicyclomine		p-value Fluoxetine & Duloxetine Hydrochloride	
		Number	Percentage	Number	Percentage	Number	Percentage		0.024 0.000
Abdominal pain duration before intervention	More than 9 days	11	18.0	10	16.4	11	15.0		
	Between 6 and 9 days	27	44.3	27	44.3	24	47.5		
	Between 3 and 6 days	23	37.7	21	34.4	19	35.0		
	Between 0and 6 days	0	0	3	4.9	6	2.5		
Abdominal pain duration after intervention	More than 9 days	0	0	1	1.6	5	8.3		
	Between 6 and 9 days	13	21.3	6	9.8	6	10		
	Between 3 and 6 days	32	52.5	24	39.3	20	33.3		
	Between 0and 6 days	16	26.2	30	49.2	29	48.3		
	p-value	0.002		<0.0001		0.480			
	p-value	0.003							

**Table 5:** The effects of the studied drugs on Lifestyle disruption

Variable		Fluoxetine		Duloxetine Hydrochloride		Dicyclomine		p-value Fluoxetine and Duloxetine Hydrochloride	
		Number	Percentage	Number	Percentage	Number	Percentage	0.094 0.032	
Lifestyle disruption before intervention	Completely disrupted	9	14.8	6	9.8	11	15.0		
	Often disrupted	36	59.0	36	59.0	24	47.5		
	Often ineffective	15	24.6	19	31.1	19	35.0		
	Completely ineffective	1	1.6	0	0	6	2.5		
Lifestyle disruption after intervention	Completely disrupted	0	0	0	0	5	8.3		
	Often disrupted	13	21.3	8	13.1	9	7.2		
	Often ineffective	38	62.3	35	57.4	25	47.2		
	Completely ineffective	10	16.4	18	29.5	21	37.3		
	p-value	0.046		<0.0001		0.093			
	p-value	0.009						

## Discussion

Irritable bowel syndrome (IBS) is a functional bowel disorder characterized by a change in the 

**Table 6:** The effects of the studied drugs on Defecation frequency

Variable		Fluoxetine		Duloxetine Hydrochloride		Dicyclomine		p-value Fluoxetine and Duloxetine Hydrochloride	
		Number	Percentage	Number	Percentage	Number	Percentage		0.04 0.001
Defecation frequency before intervention	1 time	34	55.7	24	39.3	13	21.8		
	3 times	27	44.3	37	60.7	37	61.6		
	More than 3 times	0	0	0	0	5	8.3		
	Daily	0	0	0	0	5	8.3		
Defecation frequency after intervention	1 time	12	19.7	29	47.5	5	8.3		
	3 times	28	45.9	29	47.5	21	35		
	More than 3 times	15	24.6	3	4.9	23	38.3		
	Daily	6	9.8	0	0	11	18.4		
	p-value	0.043		<0.0001		0.526			
	p-value	0.004							

frequency and/or consistency of bowel movements and abdominal pain in the absence of recognizable structural abnormalities (12). About 9%-23% of the world’s population have symptoms of IBS (13). Symptoms change over time and can sometimes impair quality of life (QoL) and contribute to the high cost of healthcare (14). Treatment of IBS varies based on clinical symptoms; if the predominant symptoms are constipation, medications such as psyllium, lactulose, sorbitol, and SSRI/SNRIs should be considered. Among them, SSRI/SNRIs drugs, specifically, are concerned when patients not only continue having symptoms after applying conventional medicines, but also show dissatisfaction with them. In other words, because there is a wealth of evidence demonstrating a central nervous system disorder in this disease, the use of antidepressant treatment as an alleviative agent would probably be crucial (15-17). Therefore, the current clinical trial study aimed to evaluate the effectiveness of two SSRI (fluoxetine) and SNRI (duloxetine hydrochloride) drugs along with dicyclomine compared to using dicyclomine alone (drugs without antidepressant features but conventional for treating IBS) on patients with irritable bowel syndrome with constipation (IBS-C) (7). In the majority of patients with IBS-C, duloxetine hydrochloride was connected with considerable reductions in several IBS symptoms simultaneously with enhanced ratings of functioning and QoL. The current findings suggest that duloxetine hydrochloride may ameliorate IBS separately from its antidepressant effects, owing to the fact that as much as was possible, patients with concurrent obvious depression were not included in this study.

After the complete treatment, duloxetine hydrochloride had a far more significant effect on reducing the frequency and intensity of bloating in IBS-C patients compared to fluoxetine; conversely, dicyclomine showed no effect. In a study by Vahedi et al., fluoxetine was similarly more influential than a placebo in reducing abdominal discomfort, relieving bloating, and stool consistency, but it increased bowel movements (18). Another study by Frootan et al. on 173 IBS patients in Tehran reported that fluoxetine increased the frequency of defecation in patients with flatulence but was useless in diarrhea patients (19). These results were well-matched with the current findings, yet no study related to duloxetine’s mechanism of action or which drug acts better was found. The results further signified that the brain-gut axis generally changes visceral afferent pathways and then sensitivity to pain through various mechanisms. One of the mediators of these pathways is the corticotrophin-releasing factor (CRF) that has continuous activity when the hypothalamic-pituitary-adrenal (HPA) is disrupted. CRF's permanent activity is to release proinflammatory cytokines that result in visceral hypersensitivity and symptoms of flatulence. Therefore, using such drugs could be beneficial from this aspect (1). Grover et al. suggested that fluoxetine could help constipation-predominant symptoms by reducing orocecal transit time. Furthermore, it tends to be effective in treating constipation by shortening intestinal transit time (6, 16). Consequently, we have presented herein for the first time that duloxetine hydrochloride may have plenty of beneficial impacts on decreasing flatulence in IBS-C patients, but the underlying reason for that remains to be discovered.

As the harmful influences of IBS symptoms on daily performance and QoL are generally considered significant, along the same vein, the current study has shown a notable improvement in the QoL of those who received duloxetine hydrochloride in comparison to fluoxetine (20). In studies by Brennan and Kaplan et al., similarly, when patients with IBS were treated with duloxetine hydrochloride, many of their IBS symptoms were significantly reduced in conjunction with better QoL(8, 21) . In addition, the results of treatment with SSRI on IDB-D patients were compatible with the current findings (22). Another study conducted by Mikocka-Walus et al. closely followed the current results; they saw short-term effectiveness of duloxetine hydrochloride on enhancing QoL and anxiety but were in conflict, as fluoxetine indicated no advantage on these patients (23). This undeniable discrepancy could be justifiable with the sample size; the current study included 182 patients, whereas only 26 participants were considered in their studies. Nonetheless, the fundamental rationale for this difference needs to be taken into consideration in the future.

The current study also found that duloxetine hydrochloride significantly increased the frequency of defecation and diminished the duration and severity of abdominal pain more than fluoxetine in patients with IBS-C. In their review study, Bradesi et al. showed that SSRIs increased the frequency of defecation (24).Kaplan and Vahedi et al. approved the effect of these two drugs on reducing pain in IBS patients. Moreover, Chey and Wall et al. observed a substantial decrease in abdominal pain with duloxetine. Other studies have indicated that paroxetine (a member of the SSRI family) is useful in controlling abdominal pain and constipation symptoms in IBS patients. The pain killer role of duloxetine hydrochloride is played by it triggering inhibitory presynaptic a2 adrenergic receptors in the locus ceruleus (LC) (8). Although the current findings were proven by the aforementioned studies, none of them compared these two drugs (18, 21 25-28). In contrast, a study carried out by Kuiken et al. showed that fluoxetine was ineffective in relieving abdominal pain. This contradiction could be justified by the small size of that study (40 patients versus 180 patients in the current study) as well as the longer course of treatment (by two weeks) in the current study (29). Similar to this study, previous studies have approved the positive influence of fluoxetine on the duration of pain (3). Of note, the current results present this relationship firsthand over and above these findings that neither gender nor age affected the amount of pain that patients tolerate after treatment with these types of drugs. 

In a meta-analysis study performed by Friedrich, patients were treated with anti-depression drugs such as duloxetine hydrochloride and mirtazapine. They concluded that such treatments could be advantageous for IBS patients; the current study was in agreement with these results, but herein, each symptom of IBS was studied, and patients did not suffer from severe depression (1).

According to the current results, the therapeutic effects of fluoxetine/dicyclomine and duloxetine hydrochloride/dicyclomine on all symptoms of IBS-C were greater than the placebo (dicyclomine), and duloxetine hydrochloride was able to reduce symptoms more than fluoxetine, in contrast to what has been considered as a side effect for this drug. Notwithstanding the fact that the dominant mechanism of action of these drugs in IBS-C patients remains to be elucidated, we are assured, based on this and other studies, that duloxetine hydrochloride acts independently of its antidepressant effects, as individuals with concurrent depression were excluded (8). Further studies to clarify some vagueness should be considered. These include, but are not limited to, what side effects do these drugs have? What are the best dosage and length of treatment? What are the main mechanisms of these drugs? Will these anti-depression drugs be effective if they are used solely without conventional medicines such as dicyclomine? By answering these questions, these drugs, with duloxetine hydrochloride as a priority, could be recommended for use in patients with IBS-C in the future. Additionally, as they are against the overuse of other drugs, they may result in less harm to patients and reduced treatment costs.

## Conflict of interests

The authors declare that they have no conflict of interest.
